# Giant Phonon Anharmonicity and Anomalous Pressure Dependence of Lattice Thermal Conductivity in Y_2_Si_2_O_7_ silicate

**DOI:** 10.1038/srep29801

**Published:** 2016-07-19

**Authors:** Yixiu Luo, Jiemin Wang, Yiran Li, Jingyang Wang

**Affiliations:** 1 High-performance Ceramics Division, Shenyang National Laboratory for Materials Science, Institute of Metal Research, Chinese Academy of Sciences, 110016 Shenyang, China

## Abstract

Modification of lattice thermal conductivity (*κ*_*L*_) of a solid by means of hydrostatic pressure (*P*) has been a crucially interesting approach that targets a broad range of advanced materials from thermoelectrics and thermal insulators to minerals in mantle. Although it is well documented knowledge that thermal conductivity of bulk materials normally increase upon hydrostatic pressure, such positive relationship is seriously challenged when it comes to ceramics with complex crystal structure and heterogeneous chemical bonds. In this paper, we predict an abnormally negative trend *dκ*_*L*_/*dP *< 0 in Y_2_Si_2_O_7_ silicate using density functional theoretical calculations. The mechanism is disclosed as combined effects of slightly decreased group velocity and significantly augmented scattering of heat-carrying acoustic phonons in pressured lattice, which is originated from pressure-induced downward shift of low-lying optic and acoustic phonons. The structural origin of low-lying optic phonons as well as the induced phonon anharmonicity is also qualitatively elucidated with respect to intrinsic bonding heterogeneity of Y_2_Si_2_O_7_. The present results are expected to bring deeper insights for phonon engineering and modulation of thermal conductivity in complex solids with diverging structural flexibility, enormous bonding heterogeneity, and giant phonon anharmonicity.

Phonon engineering, in terms of controlling heat flow within the lattice by manipulating phonon behavior, has became a powerful paradigm in the design and optimization of advanced materials with optimal thermal conductivity. The key concern is to modulate phonon transport by modifying different phonon scattering mechanism, including intrinsic phonon-phonon scatterings, and extrinsic scatterings by boundary and point defects^1^. In fact, significant reduction of thermal conductivity has been realized in a wide range of energy conservation technologies via material design strategies such as doping, nanostructuring and synthesis of superlattice^2^,^3^,^4^,^5^. However, the intricacy and uncertainty of these methods leaves open the necessity of searching for effective phonon engineering methodology based on manipulation of intrinsic structural characteristics.

Over the years, tailoring thermal transport in solids by applying hydrostatic pressure has been an attractive subject of experimental and theoretical investigations in a broad variety of materials from thermoelectrics and thermal insulators to minerals in mantle, because the phonon behaviors, which provide the detailed profile of lattice thermal conduction, is sensitive to volume change of the unit cell^6^. In a majority of materials, including diamond^7^, alkali halides^8^,^9^, ice^10^,^11^ and mantle materials^12^,^13^,^14^,^15^,^16^,^17^, thermal conductivity has been found to increase with compressive pressure. Well documented knowledge in the literature states that, elevated frequency scale of phonons caused by strengthened atomic bonding tends to facilitate thermal conduction by increasing the acoustic phonon velocity and reducing the phonon scattering rate, which has long been approved as an universal feature of most solids. However, negative pressure dependence of lattice thermal conductivity (*κ*_*L*_) has also been reported in recent years, which enlightens the novel strategy of bringing down *κ*_*L*_ by applying compressive hydrostatic pressure with respect to manipulating phonon behaviors. By using first principles calculation combined with iterative solution of Boltzmann equation, Lindsay investigated the pressure dependence of *κ*_*L*_ in some III-V, II-VI cubic compounds (such as BeTe)^18^, with a characteristic of significant acoustic-optic frequency gap created by large atomic mass ratio. In this case, the separation of longitudinal and transverse acoustic phonon branches under hydrostatic pressure raises the scattering rate among them and thus leads to decreased *κ*_*L*_. Nevertheless, the reported upward shift of each phonon branch to higher frequency range under pressure still complies with the traditional cognition, mainly due to the simple crystal structure and weak bonding heterogeneity of these studied binary compounds. Using similar methodology, Ouyang^19^ observed a *dκ*_*L*_/*dP *< 0 relationship in HgTe with partially ionic atomic bonds, in which the reduction of relaxation time of transverse acoustic phonons considerably overwhelms the less enhancement of group velocity of longitudinal acoustic and optic modes. Such mechanism, however, cannot directly guide the investigations of complicated-structured ceramics with multiple chemical compositions and covalent and heterogeneous bonding features.

Recently, our density functional theoretical (DFT) calculations on high-pressure Raman spectroscopy of γ-Y_2_Si_2_O_7_ (referred as Y_2_Si_2_O_7_ for brevity) have identified obvious softening of low-frequency optic phonon modes. And this is much likely to account for significant acoustic-optic coupling, which has been recognized in other complex-structured materials with similar structural characteristics^20^,^21^,^22^,^23^,^24^. Intuitively, it is necessary to explore how this abnormal pressure-induced softening of optic phonons would affect *κ*_*L*_. The unexpected results might cast light on tuning *κ*_*L*_ with respect to modulating the scattering mechanism among phonons, as well as the group velocity of acoustic phonons. Besides, considering the promising application of Y_2_Si_2_O_7_ as high-temperature environment/thermal barrier coatings (ETBC)^25^, a comprehensive understanding of *κ*_*L*_ on the basis of phonon engineering bares its values in both scientific understanding and technological application. In a broader perspective, the interlaced stacking of rigid [SiO_3_-O-SiO_3_] units (or referred to as Si_2_O_7_ where two adjacent SiO_4_ tetrahedra are bridged by an central O atom in a Si-O-Si bond) and soft YO_6_ octahedra in Y_2_Si_2_O_7_ makes it a desirable model system for materials consisting of structural units in diverging flexibility, which covers large varieties of ternary/quaternary oxides.

In this study, we investigate the phonon dispersion and anharmonicity, and lattice thermal conductivity of Y_2_Si_2_O_7_ at various compressive hydrostatic pressure using DFT calculations. The signature of a giant acoustic-optic coupling is identified with the character of some low-lying optic phonons interacting with acoustic phonons. Unexpected anomalous decrement of *κ*_*L*_ is observed in pressured lattice; and the physical mechanism is disclosed as combined effects of 1) slightly decreased acoustic phonon group velocity and 2) significantly augmented scattering of acoustic phonons owing to pressure-induced enhancement of phonon anharmonicity. Additionally, we find that longitudinal acoustic phonons are more effective heat-carriers in Y_2_Si_2_O_7_ and dominate the magnitude of decrement of total *κ*_*L*_ under pressure; whereas transverse acoustic phonons are more intensely scattered by low-lying optic phonons and yield negligible contribution to thermal conduction.

## Results

The crystal structure of Y_2_Si_2_O_7_ remains stable up to the hydrostatic pressure of *P* = 3.06 GPa, equivalent to a volume contraction of 2%. In practice, the structural stability is guaranteed by steady changes of lattice parameters, bonding characteristics, configuration of structural units and elastic parameters. Pressure dependences of lattice constants and atomic bond lengths (See Supplementary Fig. S1 for details) indicate that deformation pattern of Y_2_Si_2_O_7_ under hydrostatic pressure involves coordinated contraction of both Si-O and Y-O structural units, leading to packing densification. Besides, based on a statistical analysis on the configuration of specific Si_2_O_7_ and YO_6_ polyhedra, we find that the average length of Y-O bonds changes from 2.240 Å at *P* = 0 GPa to 2.225 Å at *P* = 3.06 GPa, whilst that of Si-O bonds changes from 1.626 Å to 1.621 Å. Thus, the variation percentage is larger for Y-O (0.67%) than Si-O (0.31%) bonds. Similarly, the angle of intra-octahedral O-Y-O bonds changes by as much as 1.01%, larger than that of intra-tetrahedral O-Si-O (no more than 0.64%). Evidently, YO_6_ octahedron deforms in higher magnitude under pressure as if it is more “flexible” than Si_2_O_7_ unit, and actively undergoes localized distortion to accommodate the loaded strain. Such unique response of structural feature to hydrostatic pressure will be further manifested in elastic properties, which will be discussed later.

### Phonon dispersions - Giant acoustic-optic coupling in Y_2_Si_2_O_7_

**Figure 1:**
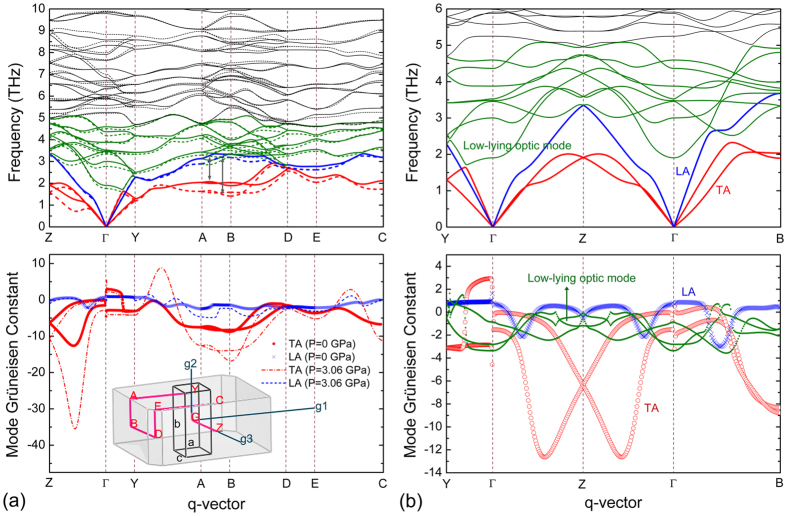
Results of lattice dynamics calculation. (**a**) Phonon dispersions (below 10 THz) of Y_2_Si_2_O_7_ at *P* = 0 GPa (solid line) and *P* = 3.06 GPa (dashed line) (i.e. TA, LA, low-lying optic and high-lying optic phonons are highlighted in red, blue, green and black lines, respectively; and grey arrows measure the magnitude of LA-TA separation) and corresponding mode Grüneisen constant (*γ*_*i*_(*q*)) of acoustic phonons. (Inset) First Brillouin zone with high-symmetry points considered in our phonon dispersion calculations. (**b**) Phonon dispersion (below 6 THz) of Y_2_Si_2_O_7_ at *P* = 0 GPa along principal directions of reciprocal lattice; and corresponding mode Grüneisen constant (*γ*_*i*_(*q*)) zoomed in for acoustic and three low-frequency optic phonons.

Phonon dispersion curves along high-symmetry directions in Brillouin zone (BZ) are calculated at different pressure, and the results of two typical cases (*P* = 0 and 3.06 GPa) are plotted in Fig. 1(a). A general feature is the highly mixing of low-frequency optic phonons and longitudinal acoustic (LA) phonons at multiple BZ points, indicative of a giant acoustic-optic coupling in Y_2_Si_2_O_7_ due to the close-up of acoustic-optic frequency gap. Particularly, a striking observation is the avoided crossing^26^ between the lowest optic phonons (ν ~ 1.9 THz at Г point) and transverse acoustic (TA) phonons, seen as a repulsive trend between the two groups of curves. To explicitly elucidate this phenomenon, phonon dispersions (below 6 THz) of the equilibrium structure along the principal directions of reciprocal lattice are presented in Fig. 1(b), together with corresponding mode Grüneisen constant (*γ*_*i*_(*q*)) curves zoomed in for acoustic and three low-frequency optic phonons. As is depicted, the lowest optic phonon branch exhibits an apparent drop into the acoustic phonon regime at around the BZ center, and results in either flattening out or curving back of TA modes at approximately midway to the BZ boundary, leading to abrupt changes of calculated *γ*_*TA*_(*q*) due to avoided crossing between TA and low-lying optic phonons. On comparison, LA mode could better retain its feature towards the BZ boundary without drastic softening; and accordingly, calculated *γ*_*LA*_(*q*) curve exhibits as some small bumps, originated when LA cross the low-lowing optic phonons with slight fluctuation of its slope. Typically, optic phonons do not directly participate in thermal transport process due to the low group velocity, yet they provide essential scattering channels for heat-carrying acoustic phonons. Therefore, one could expect that the coupling between low-lying optic phonons and TA/LA phonons should play an important role in determining the thermal conduction in Y_2_Si_2_O_7_. One thing to notice is the much higher absolute values of *γ* for TA (above ~10) than those for LA (within ~3) phonons especially along Г→Z path; which, based on the 1/*γ*^2^ dependency of phonon relaxation time^27^,^28^, indicates that TA phonons have considerably higher anharmonicity and undergo stronger scattering than LA phonons.

It is shown in Fig. 1(a) that under pressure of *P* = 3.06 GPa, the majority of high-lying optic phonons are raised to higher frequencies as a result of strengthened atomic bonding, analogous to the general trend in most solids. In contrast, the collective of low-lying optic and acoustic phonons shift downwards across entire BZ, which is an unconventionally interesting phenomenon; accordingly, *γ*_*i*_(*q*) (<0 for most points) for all three acoustic modes moves to higher absolute values, indicative of enhanced acoustic phonon anharmonicity. Further comparison among acoustic phonons indicates that TA phonons are more susceptible to hydrostatic pressure than LA phonons, reflected by the higher magnitude of downward frequency shift as well as higher enhancement of *γ*_*TA*_(*q*) values. Here, we note that the softening of low-frequency phonons under hydrostatic pressure is a direct manifestation of the localized accommodation of applied strain, rather than the softening of entire lattice that implies structural instability.

### Pressure dependence of intrinsic lattice thermal conductivity

**Figure 2:**
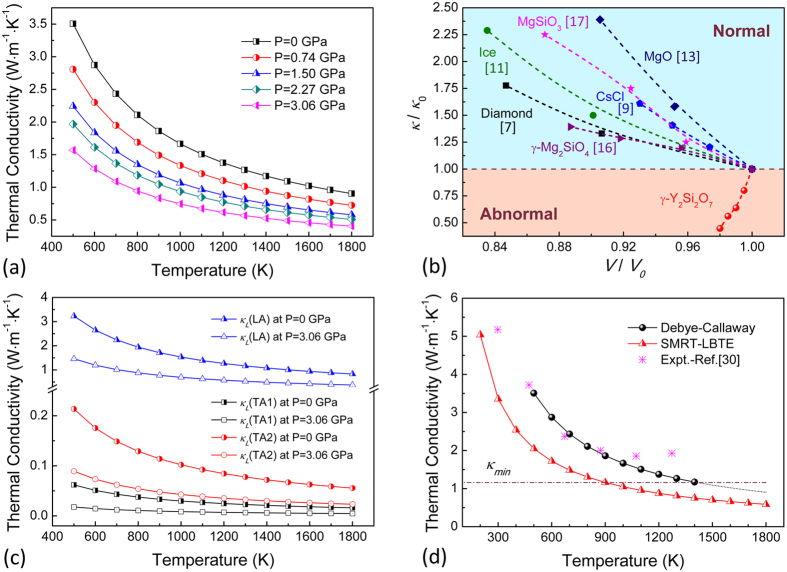
(**a**) Temperature dependence of lattice thermal conductivity (*κ*_*L*_) for Y_2_Si_2_O_7_ as a function of hydrostatic pressure up to *P* = 3.06 GPa. (**b**) Comparison of normal and abnormal *κ*_*L*_(*P*) dependency in some representative solids. Referenced data are collected at 300 K for diamond^7^ and Ice^11^, 296 K for CsCl^9^, 2000 K for MgO^13^, 1000 K for γ-Mg_2_SiO_4_^16^ and MgSiO_3_^17^, and 600 K for Y_2_Si_2_O_7_. *κ*_*0*_ and *V*_*0*_ denote lattice thermal conductivity and equilibrium volume, respectively, at *P* = 0 GPa for each referenced compound. (**c**) Partial contributions (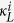) of TA and LA phonons to the total *κ*_*L*_ of Y_2_Si_2_O_7_ at *P* = 0 and 3.06 GPa (**d**) Comparison of lattice thermal conductivity of Y_2_Si_2_O_7_ (*P* = 0 GPa) from modified Debye-Callaway model, solution of linearized phonon Boltzmann equation combined with single-mode relaxation-time approximation (SMRT-LBTE method), and experimental data^30^. Minimum thermal conductivity (*κ*_*min*_)^52^ at equilibrium geometry is presented in dash-dot line as a guide for the eye.

Intrinsic lattice thermal conductivity of Y_2_Si_2_O_7_ is calculated as a function of hydrostatic pressure based on modified Debye-Callaway model^27^,^28^. As is presented in Fig. 2(a), *κ*_*L*_ of Y_2_Si_2_O_7_ monotonously decreases upon hydrostatic pressure over the calculated temperature range; and the remarkable decrement (55.2%) of *κ*_*L*_ at 600 K (around Debye temperature of Y_2_Si_2_O_7_, 575 K) sharply contradicts that of other referenced materials in Fig. 2(b)^7^,^8^,^9^,^10^,^11^,^12^,^13^,^14^,^15^,^16^,^17^. We also investigate the partial contributions of TA and LA branches to the total *κ*_*L*_ and results are plotted in Fig. 2(c) for the end-pressure cases (*P* = 0 and 3.06 GPa). Of great interest, LA phonons contribute as much as 90% to the total *κ*_*L*_ within studied temperature (pressure) range, declaring its overwhelmingly effective role over TA phonons in thermal conduction of Y_2_Si_2_O_7_. In fact, the trivial contribution of TA phonons could be fundamentally explained by stronger scattering with low-lying optic phonons, as we have observed from phonon dispersion curves. And such phenomenon has also been found in rare-earth pyrochlores with stacked polyhedra units^29^. Consequently, even though the partial 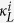 of TA phonons decreases in higher proportion than LA (~71% decrement for 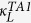, 58% for 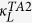 and 55% for 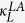 at *P* = 3.06 GPa), it is the pressure dependence of LA phonons that dominates the abnormal tendency as well as the magnitude of *dκ*_*L*_/*dP *< 0 relationship in Y_2_Si_2_O_7_.

In order to validate the reliability of modified Debye-Callaway model used in the present study, we herein compare our predicted *κ*_*L*_ of Y_2_Si_2_O_7_ with experimental data^30^ measured for polycrystalline samples at ambient pressure. As is shown in Fig. 2(d), *κ*_*L*_ calculated from present model manages to capture the *κ*_*L*_ ~ 1/*T* dependency at around and above Debye temperature where Umklapp phonon scattering process dominates, and generally agrees well with experimental data. The slight discrepancy is understandable considering the fact that experimental data normally renders an over-flattened *κ*_*L*_-*T* relationship, i.e. defects contained in polycrystalline experimental samples might serve as effective phonon scatters at relatively low temperature regime in addition to the intrinsic Umklapp phonon-phonon scattering; while high-temperature measurements are susceptible to thermal radiation, which brings extra heat and thus result in higher or even ascending experimental values at high temperature region. Besides, we also calculate *κ*_*L*_ of Y_2_Si_2_O_7_ at a typical case (*P* = 0 GPa) from the combination of single-mode relaxation-time approximation and a full solution of linearized phonon Boltzmann equation (SMRT-LBTE), using DFT-calculated harmonic and anharmonic interatomic force constant (IFC) as inputs^31^. However, calculated *κ*_*L*_ is seriously underestimated, which is a widely observed trend of this method originated from its basic methodology by taking phonon-phonon Normal scattering process as an independent channel for thermal resistance^32^. From above comparison, we could find that modified Debye-Callaway model is sufficiently reliable to reproduce the temperature dependence of *κ*_*L*_ for Y_2_Si_2_O_7_.

**Figure 3:**
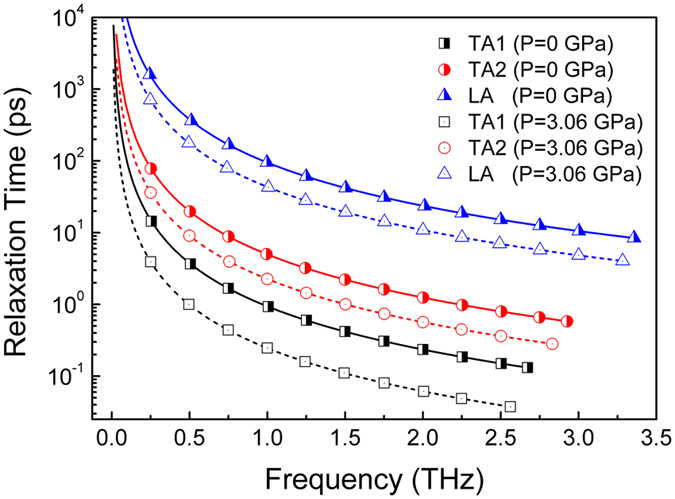
Calculated frequency-dependent phonon relaxation time 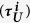 of each acoustic phonon branch at *T* = 600 K for equilibrium (*P* = 0 GPa) and pressured (*P* = 3.06 GPa) Y_2_Si_2_O_7_.

Elucidating the driving force of the abnormal *dκ*_*L*_/*dP *< 0 in Y_2_Si_2_O_7_ requires explicit examination of decisive phonon transport parameters, as listed in Table 1. Here, parameters are directly extracted from phonon dispersions; and different phonon branches are sorted according to their frequencies and eigenvectors. Firstly, values of *θ*_*i*_, which designate the maximum frequency for each phonon branch, slightly decrease with applied pressure. Besides, the average acoustic group velocity is calculated by averaging over three acoustic phonon branches as 
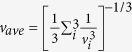
, and yields a 9.5% decrement up to *P* = 3.06 GPa. These results are consistent with the observed acoustic phonon softening upon hydrostatic pressure. Another key factor entering the calculation of 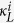 is the frequency-dependent phonon relaxation time 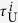, and results for three acoustic phonons calculated at a typical temperature of 600 K are plotted in Fig. 3. As we could see, TA phonons in overall have overwhelmingly smaller relaxation time than LA phonons, with orders of difference in magnitude. Plus, values of 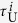 for each acoustic phonons decrease under hydrostatic pressure, up to approximately 72% for TA1, 67% for TA2 and 54% for LA phonons. According to the inverse relation between phonon scattering rate and phonon relaxation time under relaxation time approximation (RTA), we come to a vivid screenshot of the phonon transport details in Y_2_Si_2_O_7_: 1) TA phonons are strongly scattered by low-lying optic phonons, which fundamentally explains its minor contribution to thermal conduction of Y_2_Si_2_O_7_; 2) anharmonic phonon scattering is augmented under applied hydrostatic pressure, which could also be derived from tremendous increment of 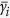 for all the three acoustic phonons. To sum up, the predicted abnormal decrement of *κ*_*L*_ in Y_2_Si_2_O_7_ is originated from significantly augmented scattering of acoustic phonons induced by enhanced phonon anharmonicity, in corporation with slightly decreased acoustic phonon group velocity.

### Pressure-induced augment of phonon scattering

In order to better understand the underlying physical mechanism of the abnormal *dκ*_*L*_/*dP* < 0 dependency in Y_2_Si_2_O_7_, we move on to specifically examine the key factor, i.e. scatterings of acoustic phonons. In dielectric crystals, the intrinsic *κ*_*L*_ is governed by phonon-phonon scatterings rising from anharmonicity of interatomic potential^33^, and the most important resistive three-phonon scatterings involves two acoustic phonons combining with one optic phonon (aao) or with another acoustic phonon (aaa)^34^,^35^. Naturally, one could expect that the frequency change of both low-lying optic and acoustic phonons might bring significant modulation to different three-phonon scattering options, and thus determines heat transport process. It is shown in Fig. 1(a) that under *P* = 3.06 GPa, the overall decreased frequency scale within the lower part of phonon spectrum (ν ≦ 5 THz) increases the populations of low-lying optic and acoustic phonons^36^; and meanwhile, the severe softening of TA phonons leads to a lower degree of “acoustic bunching” seen as the separation of TA further away from LA phonons, illustrated by grey arrows in Fig. 1(a). These features, to a qualitative extent, indicates an enlarged phase space for both (aao) and (aaa) scattering options^18^,^34^,^35^,^36^,^37^, and thus points to a higher phonon scattering rate. Here we note that, such speculation based on analysis of phonon dispersion could explain the enhanced phonon scattering under pressure; nevertheless, future investigations (e.g. on energy-momentum surfaces, phonon scattering channels or phase space)^38^ are required to fully clarify the interplay of different scattering options involving low-lying optic and acoustic phonons in complex ceramics, as well as its evolution upon modulation of structural features.

### Structural origin of low-lying optic phonons and giant phonon anharmonicity

**Figure 4:**
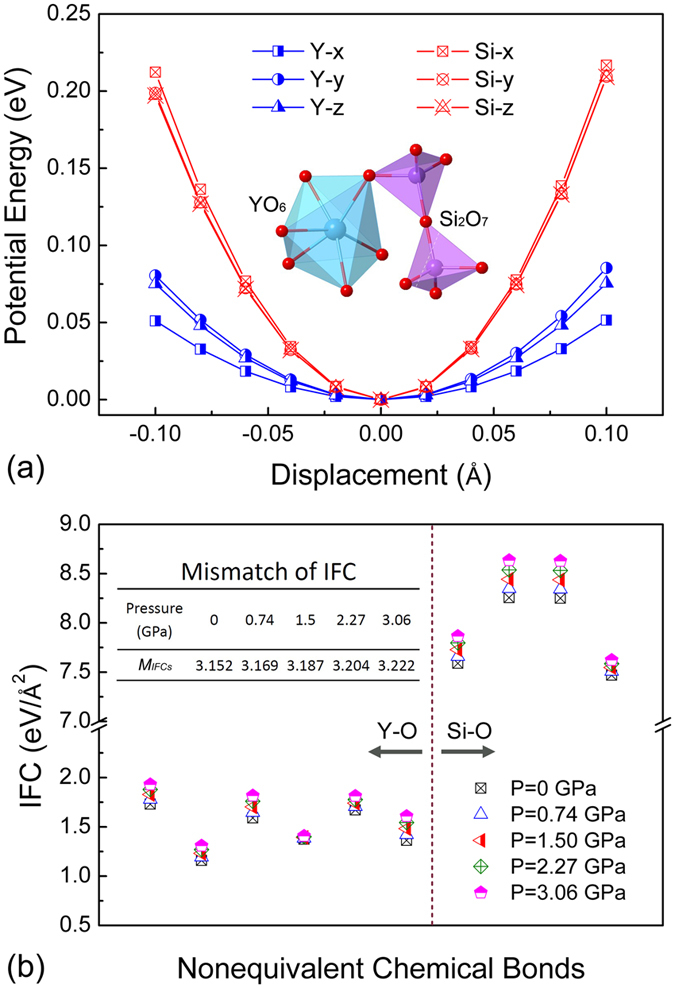
(**a**) Calculated potential energy curves for Y and Si atoms (along x-, y- and z- directions in Cartesian coordination) as a function of displacement around the equilibrium atomic positions. (Inset) Sketch of [SiO_3_-O-SiO_3_] and YO_6_ structural units in the lattice. (**b**) Calculated second-order interatomic force constant (IFC) of neighboring atomic bonds in Y_2_Si_2_O_7_ as a function of pressure. Left and right panel correspond with nonequivalent Y-O and Si-O bonds, respectively. (inset) Calculated mismatch of force constant (*M*_*IFCs*_) for Y_2_Si_2_O_7_ under different pressure; and samples used in the statistical analysis consist of ten nonequivalent chemical bonds (four Si-O and six Y-O) in the unit cell.

Clearly evidenced from above results, the low-lying optic phonons play a significant role in determining the thermal conductivity of Y_2_Si_2_O_7_ as well as its pressure dependency, through severe coupling with heat-carrying acoustic phonons. Analysis of mode eigenvectors reveals that the low-lying optic phonons (with ν ≦ 5 THz) correspond with low-energy translation of Y atoms, coordinated by tilting or bending of rigid [O_3_Si-O-SiO_3_] units. Distinguishment of the eigenvectors of these optic phonons from those lying in higher frequency range is presented in Supplementary Discussion and Supplementary Fig. S2. In fact, such unique vibration pattern is originated from the specific heterogeneity of interatomic environment in Y_2_Si_2_O_7_. To better elucidate this point, potential energy curves of Y and Si atoms are calculated by shifting the atoms away from their static equilibrium positions and calculate the energy difference with respect to their equilibrium state. As is shown in Fig. 4(a), Y atom (in heavier mass) sits in a very flat potential well and is loosely bonded with surrounding O atoms. In contrast, the potential well of Si atom (in lighter mass), which forms much stronger chemical bonds with the surrounding atoms, is over twice steeper than that of Y. As a result, the translation of Y atom could be easily exited with lower energy, coordinated by low-energy tilting or bending of [O_3_Si-O-SiO_3_] bridged units, during which the configuration of end SiO_4_ tetrahedra shows negligible changes. What worthy of mention is that, the inherent correlation between interatomic bonding heterogeneity and low-frequency optic phonons, contributed by coupled vibration of a set of atoms and thus run over a wide range in the lower part of phonon spectrum, has also been observed in other ternary materials showing a part-crystalline part-liquid state^39^.

Plus, it would be illuminating to dig out the structural origin of the pronounced enhancement of phonon anharmonicity in pressured Y_2_Si_2_O_7_. According to an empirical model treating crystal lattice as spring network^40^, the extent of lattice anharmonicity of materials has a positive correlation with the mismatch of force constants among neighboring bonds. There are four (six) nonequivalent Si-O (Y-O) bonds in the unit cell of Y_2_Si_2_O_7_, constituting a sample of ten different chemical bonds in our study. As is shown in Fig. 4(b), IFCs of Si-O bonds are approximately three times higher than those of Y-O bonds, indicative of higher strength of Si-O than Y-O bonds; and accordingly, significant bonding heterogeneity in Y_2_Si_2_O_7_. Besides, as hydrostatic pressure increases to *P* = 3.06 GPa, IFCs for both types of bonds are raised to higher values, which is a direct reflection of the enhanced overall atomic bonding strength due to volume contraction. In order to quantify the mismatch of force constant of atomic bonding in each crystal geometry, a parameter *M*_*IFCs*_ is defined as 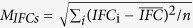 (*n* = 10 in our statistical analysis) based on the average squared deviation of IFC for *i*^*th*^ individual bonds (*IFC*_*i*_) from their arithmetic mean value (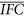). Results are summarized in the inset of Fig. 4(b). Clearly evident, the value of *M*_*IFCs*_ is gradually raised by 2.2% at *P* = 3.06 GPa. Therefore, the increased lattice anharmonicity in pressured crystal is a reasonable consequence of the enlarged mismatch of force constants between neighboring bonds.

### Correlation between elastic properties and acoustic phonon softening

It is well known that elastic moduli of a material are closely related with the long-wavelength part of the acoustic phonon branches near BZ center, and thus are critical parameters in tracing the physical properties of acoustic phonons. Calculation of elastic parameters reveals that under hydrostatic pressure, the overall strength of the structure (measured by bulk modulus *B*) is enhanced by Δ*B*/Δ*P* = 3.00, as expected from higher average atomic bonding strength upon lattice contraction; however, the resistance to shear deformation (measured by shear modulus *G*) shows a weak decrease as Δ*G*/Δ*P* = −0.57 (See Supplementary Discussion and Supplementary Fig. S3 for details). This abnormity could be attributed to active accommodation of shear strain by the localized deformation of soft and flexible YO_6_ polyhedral units, which has been reported as the typical shear deformation mechanism in yttrium silicates^41^, and again, is originated from the intrinsic bonding heterogeneity of Y_2_Si_2_O_7_. In fact, it is fundamental knowledge that average sound velocity (*v*_*m*_) could be approximated as acoustic phonon group velocity by assuming linearity of phonon dispersion, written in the form of 
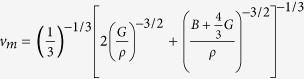
 (*ρ* being density of crystal); and hence calculated Δ*v*_*m*_/Δ*P* < 0 dependency qualitatively echoes the negative pressure dependence of *v*_*ave*_, as we have covered before. Therefore, the weakly decreased (obviously increased) *G* (*B*) value is a mirror of the pressure-induced softening of acoustic phonons in Y_2_Si_2_O_7_, and has also been observed in other materials with the complicated interpenetrating of both soft and rigid (usually tetrahedrally coordinated) structural units^42^. Accordingly, one might be able to make a primary deduction on the acoustic phonon behavior, and furthermore, the positive or negative *κ*_*L*_(*P*) relationship of a novel material by monitoring the pressure dependence of elastic moduli, which could be easily obtained from either theoretical or experimental approaches.

## Discussions

In this study, we predict an anomalous decrement (55.2%) of lattice thermal conductivity (*κ*_*L*_) in Y_2_Si_2_O_7_ under hydrostatic pressure up to *P* = 3.06 GPa. The mechanism of the *dκ*_*L*_/*dP* < 0 relationship is disclosed as the combination of slightly decreased group velocity and significantly augmented scattering of heat-carrying acoustic phonons. The coordinated vibration of flexible YO_6_ and rigid [SiO_3_-O-SiO_3_] structural units with highly diverse interatomic force constant renders the system a collective of low-lying optic phonons that strongly interact with acoustic phonons through acoustic-optic coupling; and the particularly severer interaction with TA than LA phonons makes the former (latter) negligible (dominant) contributors to thermal conduction. Under pressure, the anomalous downward shift of low-lying optic phonons and heat-carrying acoustic phonons tend to significantly raise acoustic phonon anharmonicity, and eventually block the phonon transport by slightly suppressing the acoustic phonon group velocity and facilitating three-phonon scattering process. Besides, upon tracing to experimentally-accessible elastic properties of Y_2_Si_2_O_7_, we find that the crystal undergoes an enhancement of the overall lattice strength under hydrostatic pressure; and at the same time maintains the capability to positively accommodate shear strain via localized structural deformation of flexible YO_6_ octahedra. This unique elastic deformation mechanism, governed by the intrinsically significant bonding heterogeneity, leads to a positive (weak) pressure-dependence of bulk (shear) modulus.

Based on above discussions, a guidance could be reached for the identification of solids in which loading hydrostatic pressure is an initiator to abnormally decreased lattice thermal conductivity. Such material should have diverging structural flexibility, enormous bonding heterogeneity, and giant phonon anharmonicity. And these criteria are predicted to cover numerous candidates of complex-structured oxide ceramics with interlaced stacking of rigid/soft polyhedral units, including various members of silicates, phosphates, zirconates and tungstates, to name a few.

From the perspective of phonon engineering, we would like to point out that the motivation of this paper is to open up the possibility of negatively tailoring lattice thermal conductivity of complex ceramics via hydrostatic pressure, based on modulation of intrinsic structural characteristics. And hence, it would be interesting to compare the phonon transport mechanisms in Y_2_Si_2_O_7_ to those reported in HgTe having simple crystal structure^19^. One point in common is that both work emphasize the significance of augmented phonon scattering in determining the *dκ*_*L*_/*dP* < 0 relationship due to enhanced phonon anharmonicity. However, major discrepancies expose if one look into specific phonon behaviors of the two systems by taking into consideration the diverging bonding characteristics. Firstly, the highly mixing of low-lying optic phonons in acoustic regime of Y_2_Si_2_O_7_ results in substantial scattering of TA phonons, leading to negligible contribution of TA phonons to thermal conduction as compared with LA phonons; In HgTe, however, the sizable acoustic-optic frequency gap seems to freeze out most (aao) process involving low-frequency TA phonons due to the restriction of phonon energy/momentum conservation; whereas LA phonons could decently participate in both (aaa) and (aao) scattering options, resulting in shortened relaxation time and attenuated contribution to thermal conduction. Consequently, it is the behaviors of LA (TA) phonons that dominate thermal transport process in Y_2_Si_2_O_7_ (HgTe). Secondly, a wide range of low-frequency optic and acoustic phonons in Y_2_Si_2_O_7_ undergo downward frequency shift upon hydrostatic pressure; while conventional phonon stiffening is observed in HgTe with minor exception for several TA phonon modes near BZ boundary. As a result, the decrement (increment) of phonon group velocity induced by phonon softening (stiffening) serves as a contributive (compensated) factor to the *dκ*_*L*_/*dP* < 0 relationship in Y_2_Si_2_O_7_ (HgTe). In essence, the role of acoustic-optic coupling is accentuated in Y_2_Si_2_O_7_, contributed by a collective of low-lying optic phonons, i.e. coupled vibration of a set of atoms in hierarchical neighboring atomic environment, which runs over respectively wide range of phonon spectrum. And such phenomenon is inclined to be observed in ceramics with multiple chemical composition, highly complicated crystal structure and significant bonding heterogeneity, rather than in the simple crystals, such as HgTe (in cubic lattice with two non-equivalent atoms in primitive unit cell). Therefore, future work on various material systems is encouraged to elucidate the interplay of different scattering options involving low-lying optic and acoustic phonons as well as its behavior upon modulation of structural characteristics, so as to better guide the practicability of phonon engineering.

## Methods

### Intrinsic lattice thermal conductivity: modified Debye-Callaway model

Temperature dependence of intrinsic lattice thermal conductivity (*κ*_*L*_) is calculated by following the formulism of modified Debye-Callaway model^27^,^28^, in which the total *κ*_*L*_ is written as the sum over one longitudinal (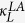) and two transverse acoustic phonons (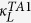 and 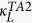); whereas the direct participation of optic phonons is neglected due to their low group velocity. And the reliability of such approximation in Y_2_Si_2_O_7_ has been verified by previous work^30^, in which relaxation time of optic phonons are found to be lower than that of acoustic phonons by 3 ~ 4 orders of magnitude.


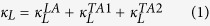


The partial conductivities 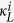 are given by:


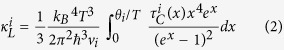


Here, *i* denotes phonon branches (LA, TA1 and TA2), *k*_*B*_ is the Boltzmann constant, 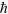 is the reduced Planck constant, *T* is absolute temperature, *v*_*i*_ is corresponding acoustic phonon group velocity, *τ*_*C*_ is the total relaxation time of phonon scattering process, 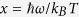 is a dimensionless quantity derived from phonon frequency *ω*; and *θ*_*i*_ is the longitudinal and transverse acoustic Debye temperature obtained from phonon dispersion as 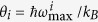, where 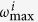 is the maximum frequency of the acoustic phonon branch *i* in each BZ direction^39^. Here, phonon group velocity *v*_*i*_ is calculated from the slope of acoustic phonon dispersion around Г point, averaged along each BZ direction.


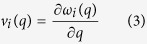


The total phonon scattering rate (*τ*_*C*_^−1^) for electrically insulating crystals normally involves contribution from Umklapp phonon scattering (*τ*_*U*_^−1^), Normal phonon scattering (*τ*_*N*_^−1^), point defect scattering due to isotopes with different mass (*τ*_*I*_^−1^), and boundary scattering (*τ*_*B*_^−1^)^27^:


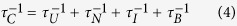


In perfect lattice, such as pure Y_2_Si_2_O_7_ in our study, the contribution from latter terms could be ignored since Umklapp phonon-phonon scattering effect dominates at above Debye temperature, and thus *τ*_*C*_ ≈ *τ*_*U*_. Although many expressions exist for *τ*_*U*_, they all take the form as quadratic inverse of *γ* with similar pre-factors^1^. Based on the work of Slack and Galginaitis^43^, we employ the following expression for the Umklapp scattering rate for phonon branch *i*^27^:


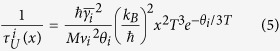


where *M* is the average mass of an atom in the crystal.

In the present calculation, mode Grüneisen constant is obtained by slightly perturbing the crystal away from the original volume *V* and calculate the resulted frequency change of each mode:


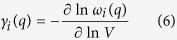


where *γ*_*i*_(*q*) and *ω*_*i*_(*q*) are mode Grüneisen constant and phonon frequency, respectively, at wave vector *q* for phonon branch *i*. Accordingly, the average Grüneisen constant 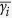 for each acoustic branch could be written as the average of *γ*_*i*_(*q*) weighed by mode contribution of specific heat capacity^29^:


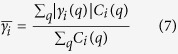



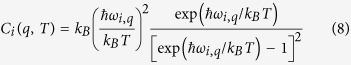


Here, the absolute value of 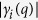 is emphasized to avoid the cancelation between modes with positive or negative values. All the parameters presented above are directly extracted from lattice dynamic calculations, allowing estimation of thermal conductivity from phonon behaviors.

As an alternative, *κ*_*L*_ could also be obtained from anharmonic lattice dynamics calculation, by solving linearized phonon Boltzmann transport equation under single-mode relaxation-time approximation (SMRT-LBTE) and employing DFT-calculated anharmonic interatomic force constants as inputs. In this method, *κ*_*L*_ takes the form of^44^:


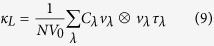


where *V*_*0*_ is the volume of a unit cell; *N* is the number of unit cells in the crystal; *λ* is abbreviated for phonon mode (*i*, *q*); *C*_*λ*_, *v*_*λ*_ and *τ*_*λ*_are the specific heat capacity, group velocity and relaxation time of mode *λ*, in regular definition as stressed before. Phonon relaxation time (*τ*_*λ*_) is computed from imaginary part of phonon self-energy, by using many-body perturbation theory with the crystal potential expanded to include third-order terms.

We should note that ceramics with low thermal conductivity (such as Y_2_Si_2_O_7_ in our study) typically has complicated crystal structure, i.e. in low crystal symmetry and contains as much as tens of atoms in the unit cell, which makes anharmonic dynamics calculation computationally formidable and time-wise unaffordable. In this situation, RTA-based Debye-Callaway model has long been approved as a convenient methodology, considering its reliability in reproducing experimental data of various ceramic systems^29^,^45^.

### Computation details

Density functional theory (DFT) calculation is performed using Vienna Ab Initio Simulation Package (VASP) program^46^,^47^, where localized density approximation (LDA)^48^ and projected augmented wave (PAW) method^49^ are employed, with a cutoff energy of 600 eV. The valence shells and electronic configurations for pseudo-atoms are 4*s*^2^4*p*^6^4*d*^1^5*s*^2^ for Y, 3*s*^2^3*p*^2^ for Si and 2*s*^2^2*p*^4^ for O. The special points sampling integration over Brillouin zone (BZ) is realized by using Γ-centered 5 × 3 × 5 Monkhorst-Pack^50^ *k*-points method. Lattice parameters and internal atomic positions are optimized until the total energy and force converge to 10^−9 ^eV and 10^−4 ^eV/Å, respectively. Calculated lattice parameters at equilibrium structure of Y_2_Si_2_O_7_ are *a* = 4.667 Å, *b* = 10.730 Å, *c* = 5.510 Å, *β* = 95.84°, which agrees well with experimental data^51^ and previous calculations^52^. The effect of hydrostatic pressure is simulated by imposing a volume contraction on the equilibrium geometry of Y_2_Si_2_O_7_ up to 2%, with an interval of 0.5%. The unit-cell vectors and atomic positions are fully relaxed via geometry optimization under each volume-fixed lattice until convergence criteria is satisfied; and the structural parameters including lattice constants, crystal symmetry, and atomic configuration are carefully monitored to ensure structural stability upon volume modulation. Lattice-dynamics calculations are carried out using the Phonopy package^53^. Second-order interatomic force constants (IFC) are calculated within the framework of density function perturbation theory (DFPT) via linear-response approach^54^. Phonon frequencies and eigenvectors are obtained from diagonalization of the dynamical matrix built by IFCs. During post-process, phonon frequencies are sampled on a Γ-centered 3 × 3 × 3 *q* mesh, which could converge the thermal dynamic properties to a sufficient extent.

SMRT-LBTE calculation is conducted using Phono3py^31^, with harmonic and anharmonic interatomic force constant extracted from DFT calculations. Calculation parameters are chosen identical as above, although 2^nd^- and 3^rd^-order IFCs are calculated from supercell approach (2 × 1 × 2 supercell containing 88 atoms) with finite atomic displacement of 0.03 Å; and translational invariance constant is enforced when dealing with 3^rd^ IFC. Atomic interaction cut-off of 3^rd^ IFC is chosen as sixth- and fourth-nearest neighbors for Y and Si atoms, respectively; whereas increasing the cut-off to include twelfth-/eighth-nearest neighbors for Y/Si renders a negligible correction (0.5%) to *κ*_*L*_. Thermal conductivity is calculated with q-mesh of 3 × 3 × 3; and a tetrahedron method^55^ is employed in calculating imaginary part of self-energy and thus phonon lifetime.

### Calculation of elastic parameters

Second-order elastic coefficients (*c*_*ij*_) are determined from the linear fit of stress as a function of applied homogeneous elastic strains^56^. We apply four homogeneous strain patterns with finite values and calculate the generated stress after optimizing the internal degrees of freedom. For each strain pattern, six amplitudes ε (three positive and three negative) are applied with |ε| ≦ 0.5%. The polycrystalline bulk modulus *B* and shear modulus *G* are determined by Voigt-Reuss-Hill (VRH) approximations^57^,^58^,^59^, where values calculated from second-order stiffness constants (*c*_*ij*_ in *C*) and second-order compliance constants (*s*_*ij*_ in *S* = *C*^−1^) are averaged to represent the real elastic moduli:


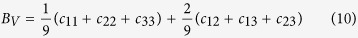



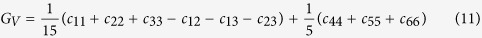



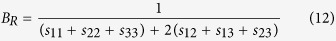



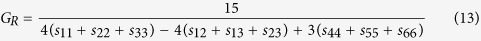



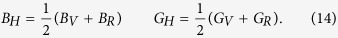


## Additional Information

**How to cite this article**: Luo, Y. *et al*. Giant Phonon Anharmonicity and Anomalous Pressure Dependence of Lattice Thermal Conductivity in Y_2_Si_2_O_7_ silicate. *Sci. Rep.* **6**, 29801; doi: 10.1038/srep29801 (2016).

## Supplementary Material

Supplementary Information

## Figures and Tables

**Table 1:** Longitudinal and transverse Debye temperatures (*θ*_*i*_), average group velocities (*v*_*i*_) and Grüneisen constants 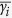 for each acoustic phonons under different simulated hydrostatic pressure, extracted from phonon dispersion.

*P* (GPa)	*θ*_*TA1*_(K)	*θ*_*TA2*_(K)	*θ*_*LA*_(K)	*v*_*TA1*_(m/s)	*v*_*TA2*_(m/s)	*v*_*LA*_(m/s)	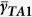	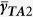	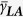
0	128	140	161	2841	4792	6930	4.976	3.827	1.367
0.74	127	139	160	2817	4818	6943	5.518	4.257	1.519
1.50	126	138	159	2791	4842	6954	6.382	4.868	1.688
2.27	124	137	158	2566	5037	6864	7.216	5.239	1.777
3.06	123	136	158	2498	5039	6865	8.357	5.867	1.976
