# Supplementary material for: Giant Phonon Anharmonicity and Anomalous Pressure Dependence of Lattice Thermal Conductivity in Y2Si2O7 silicate
Source: Sci Rep. 2016 Jul 19;6:29801. doi: 10.1038/srep29801 (PMC4949468; doi:10.1038/srep29801)
Supplement: Supplementary Information [file srep29801-s1.doc]

**Supplementary Information**

**Giant Phonon Anharmonicity and Anomalous Pressure Dependence of Lattice Thermal Conductivity in Y_2_Si_2_O_7_ silicate**

Yixiu Luo, Jiemin Wang, Yiran Li, Jingyang Wang†

High-performance Ceramics Division, Shenyang National Laboratory for Materials Science, Institute of Metal Research, Chinese Academy of Sciences, 110016 Shenyang, China

Correspondence and requests for materials should be addressed to Jingyang Wang (Email: jywang@imr.ac.cn)

**Supplementary Discussion**

***Animation of phonon eigenvector***

The eigenvectors of four optic phonons selected at Г point of Brillouin zone (BZ) are depicted by the animation in Fig. S2(a), covering the typical vibration features over low-, medium- and high-frequency ranges; and the sketch of coupled vibration pattern of SiO_4_ tetrahedron and YO_6_ octahedron under *ν*=3.46 THz and *ν*=27.26 THz is presented in Fig. S2(b). As is shown, the low-frequency optic phonon modes (*ν*=3.46 THz and *ν*=4.60 THz) involve high-magnitude translation of Y atoms, coordinated by tilting or bending of Si-O-Si bridge, during which the configuration of end SiO_4_ tetrahedra shows negligible changes. The medium-frequency optic phonon mode at *ν*=13.64 THz exhibits increased distortion of Si_2_O_7_ and YO_6_ units, where the motion of Y atoms is seriously mitigated due to their large atomic mass. And high-frequency region (represented by *ν*=27.26 THz) is dominated by stretching and serious distortion mode of Si-O and Y-O units in the lattice. The group theory predicts the irreducible representations of Y_2_Si_2_O_7_ as: 18Au+18Bu+15Ag+15Bg, where the Au and Bu (Ag and Bg) modes are IR (Raman) active. And distinction between IR- and Raman-active modes could be vividly drawn based on diverging vibration of Si-O-Si bridge, i.e. IR-active mode involves bridging O atom moving out of (mostly normal to) or along the bridge; whereas it remains still in Raman-active mode. For brevity, we only plot one IR-active mode at low-frequency region for example.

***Pressure dependence of elastic parameters***

Full set of second-order elastic constants (*c_ij_*) and polycrystalline elastic modulus are calculated as a function of pressure in our study. As is shown Fig. S3, the stiffness of crystal against principal strains (measured by *c_11_*, *c_22_* and *c_33_*) is enhanced; whereas the resistance against shear deformation (measured by *c_44_*, *c_55_* and *c_66_*) is less dependent on applied hydrostatic pressure. Accordingly, calculated polycrystalline bulk modulus (*B*) shows a monotonously increase as Δ*B*/Δ*P*=3.00, whereas shear modulus (*G*) shows a weakly negative pressure dependence as Δ*G*/Δ*P*=-0.57.

**Supplementary Figures:**


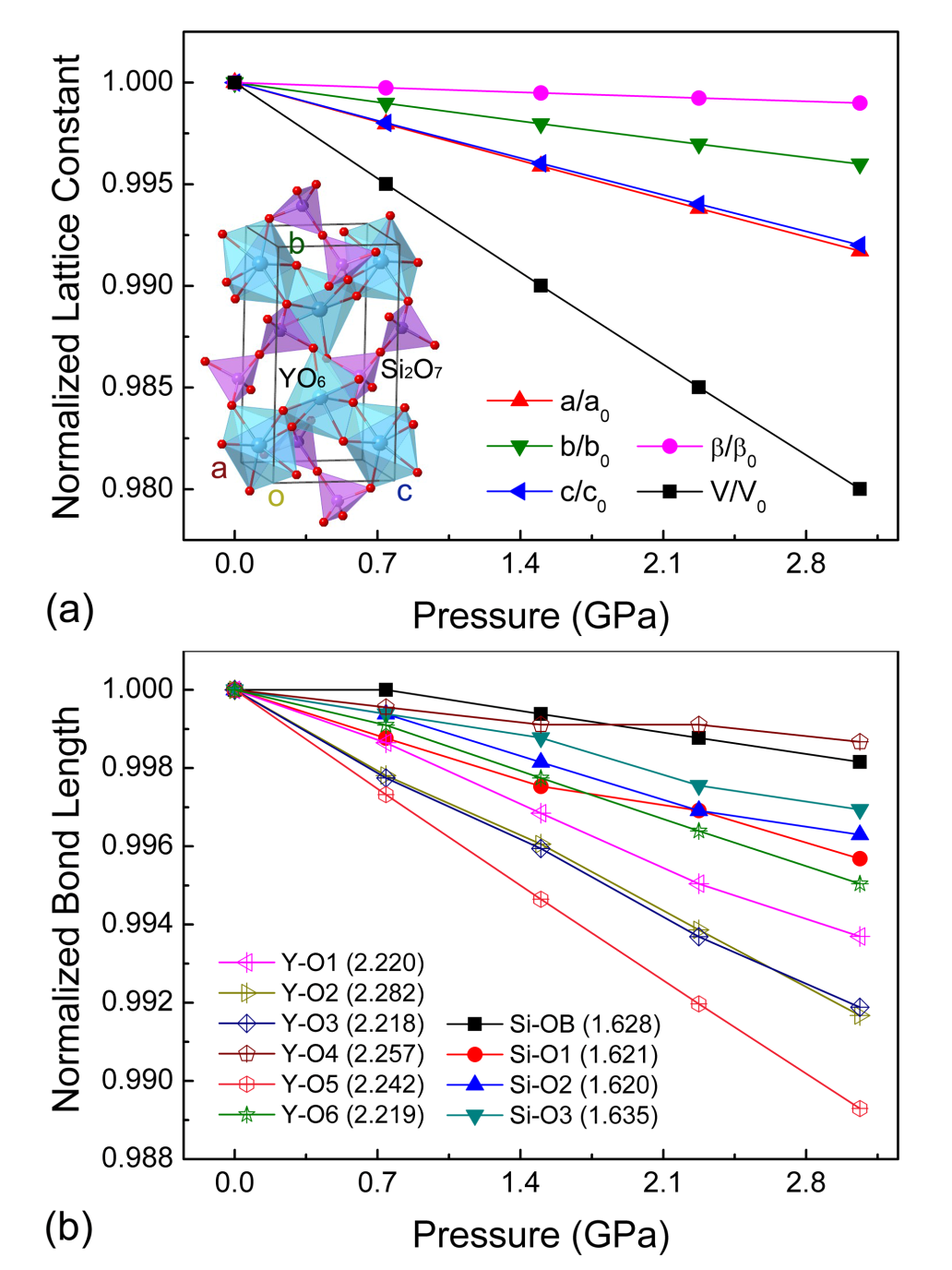


Fig. S1. Pressure dependence of structural parameters of Y_2_Si_2_O_7_. (a) Normalized lattice constants of Y_2_Si_2_O_7_ as a function of hydrostatic pressure. (Inset) Crystal structure of Y_2_Si_2_O_7_. The unit cell of Y_2_Si_2_O_7_ (space group *P*2_1_/*C*) contains 22 atoms, with one Si site, one Y site, and four different O sites. The structural characteristics could be described as rigid Si_2_O_7_ units (consist of two adjacent SiO_4_ tetrahedra forming a linear bridge by sharing a central O atom) stacking in the sandwich-like framework of YO_6_ octahedra.

(b) Normalized bond length of nonequivalent atomic bonds in Y_2_Si_2_O_7_ as a function of hydrostatic pressure. "OB" denotes the bridging O atom in the [O_3_Si-O-SiO_3_] unit. Values in bracket are bond lengths (in Å) of Si-O and Y-O in equilibrium geometry of Y_2_Si_2_O_7_, i.e. *P*=0 GPa. Under hydrostatic pressure, Y-O bonds variate in considerably higher magnitude than Si-O bonds, except for the one with medium bond length; and the bridging Si-O bond changes only slightly in comparison with other Si-O bonds.


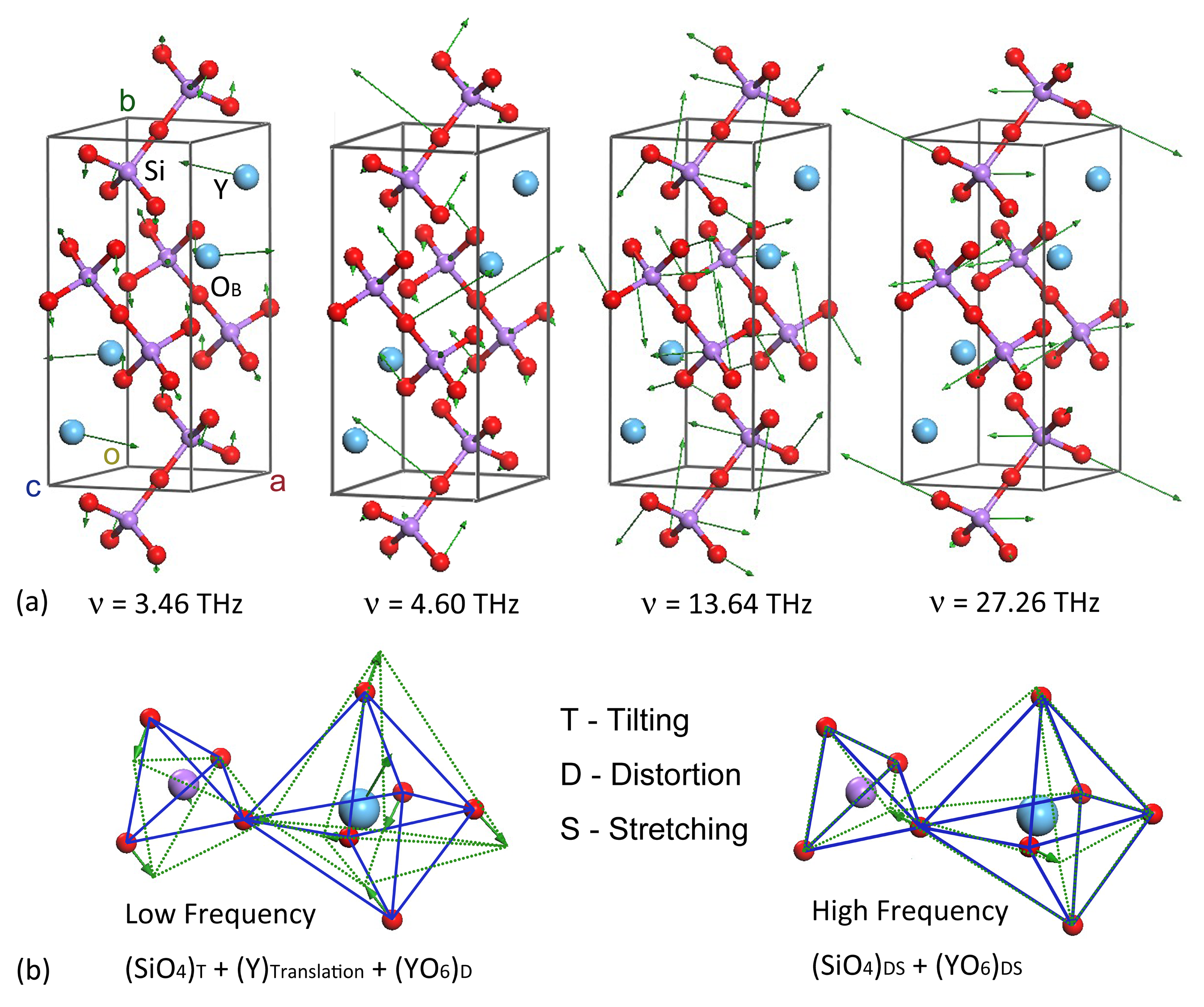


Fig. S2. Eigenvectors of phonon modes of Y_2_Si_2_O_7_ from lattice dynamics calculation at *P*=0 GPa. (a) Animation of the eigenvectors for four selected optic phonons (*ν*=3.46, 4.60, 13.64 and 27.26 THz) at Г point of Brillouin zone (BZ); and the length of green arrows is proportional to the amplitude of atomic vibration at each phonon mode. Note: for a better illustration, the length of arrows presented here is multiplied by certain factors for each phonon mode, and thus comparison between different phonon mode is meaningless.

(b) Sketch of coupled vibration pattern of SiO_4_ tetrahedron and YO_6_ octahedron under low- and high- frequencies, where solid blue and dotted green lines outline the geometric changes of Si-O and Y-O polyhedra as they vibrate around equilibrium position.


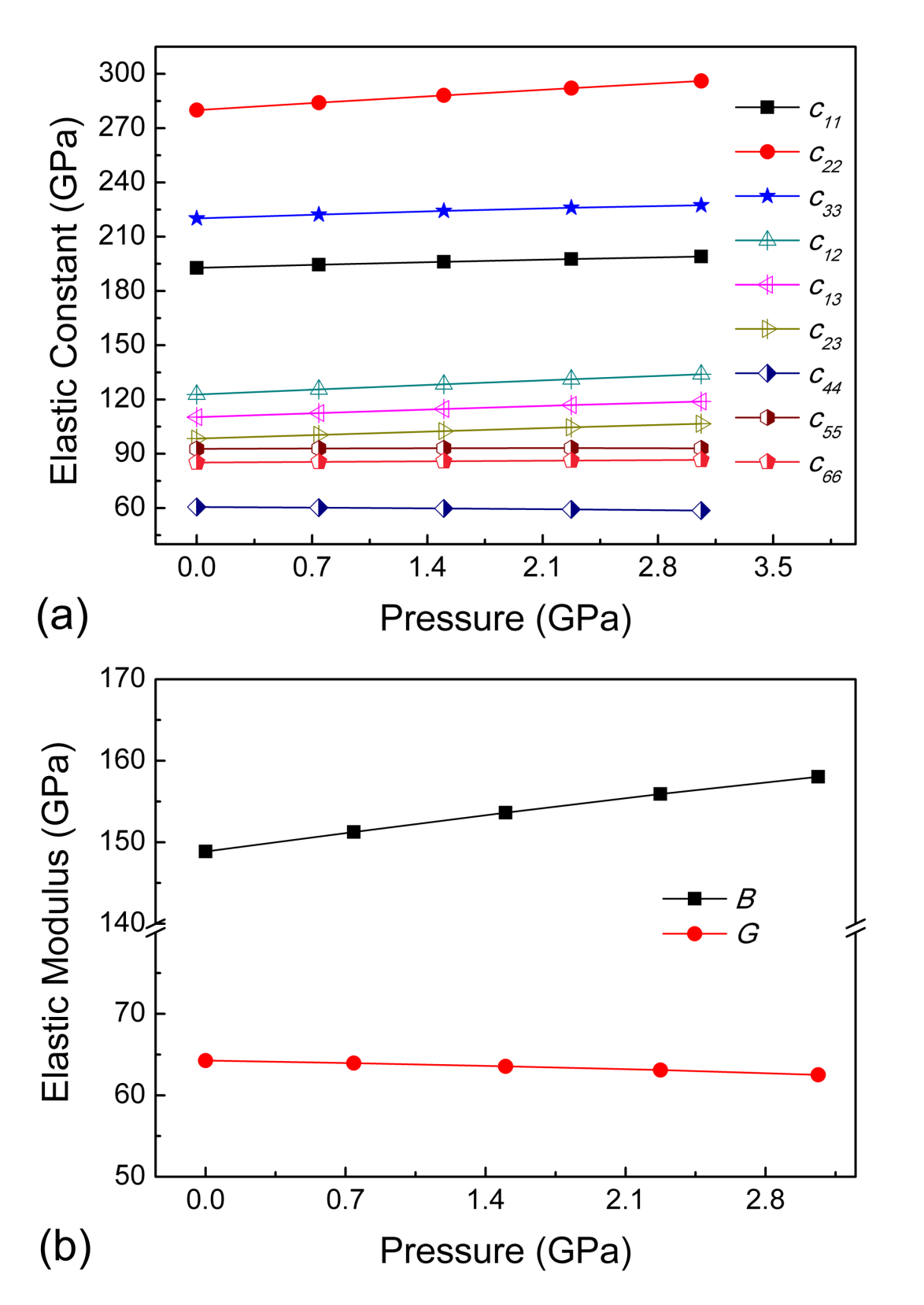


Fig. S3. Pressure dependence of calculated elastic parameters. (a) Second-order elastic constants (*c_ij_*) and (b) polycrystalline bulk modulus (*B*) and shear modulus (*G*) of Y_2_Si_2_O_7_ as a function of hydrostatic pressure.
